# Determinants of childcare service demand for infants aged 0–3 among the childbearing population in China

**DOI:** 10.3389/fpubh.2025.1624999

**Published:** 2025-07-30

**Authors:** Chun Yang, Jian Zhou, Yan Liu, Wenhui Shi, Yan Cheng, Yuxin Cao, Rui Xing, Lin Cui, Rugang Liu

**Affiliations:** ^1^Jiangsu Health Development Research Center, Nanjing, China; ^2^Jiangsu Provincial Medical Key Laboratory of Fertility Protection and Health Technology Assessment, Nanjing, China; ^3^School of Health Policy and Management, Nanjing Medical University, Nanjing, China; ^4^Laboratory for Digital Intelligence and Health Governance, Nanjing Medical University, Nanjing, China; ^5^Jiangsu Provincial Institute of Health, Nanjing Medical University, Nanjing, China; ^6^Center for Global Health, Nanjing Medical University, Nanjing, China

**Keywords:** childcare services, demand, influencing factors, children aged 0–3, childbearing age people

## Abstract

**Objectives:**

This study aims to investigate the demand for childcare services for infants aged 0–3 years among the childbearing population in China and identify its key determinants.

**Methods:**

An online survey was conducted in Suzhou, China in August 2024 using a self-designed questionnaire. Information on personal and family characteristics, as well as demand for childcare services, was collected. Descriptive statistics, chi-square tests, and binary logistic regression were used to analyze the determinants of childcare services.

**Results:**

Of 5,567 respondents, 45.9% expressed demand for childcare services for children aged 0–3 years. Binary logistic regression identified several significant predictors of demand. Notably, female gender, older age, rural residence, and lower educational attainment were associated with lower demand (*p* < 0.05). Conversely, having more children, greater trust in childcare institutions, better knowledge about childcare services, and greater awareness of childcare policies were significantly associated with higher demand (*p* < 0.05).

**Conclusion:**

Demand for childcare services was influenced by multiple factors. Enhancing subsidies and rural service accessibility, strengthening institutional credibility, implementing incentives for multi-child families, disseminating childcare knowledge and policy information and facilitating a childcare paradigm shift were recommended.

## Introduction

China’s fertility rate has experienced a sustained decline since the 1970s’ family planning policy implementation, persisting as a critical demographic challenge despite recent policy relaxations (1, 2). This demographic stagnation coincides with the disintegration of traditional multigenerational childcare models under pressures of urbanization. Contemporary Chinese women increasingly prioritize professional careers over traditional caregiving roles, creating an emergent caregiving gap partially filled by grandparents (3–5). However, empirical evidence reveals three significant limitations of this adapted grandparenting model (3). First, grandparent caregivers tend to prioritize immediate physical needs over children’s cognitive and developmental requirements, potentially compromising early childhood education outcomes. Second, impending delayed retirement policies threaten to exacerbate age-related health vulnerabilities among caregiver cohorts, raising concerns about care quality sustainability. Third, long-term excessive care for children may also harm the physical and mental health of grandparents. These structural constraints underscore the urgent need for alternative childcare solutions that reconcile workforce participation with child development imperatives - a prerequisite for fertility rate recovery.

Childcare services, defined as professional non-parental care during parental unavailability, offer potential solutions (6). Multiple studies have demonstrated the dual efficacy of childcare services in reducing family care burdens and positively influencing fertility decisions (7, 8). China’s childcare system reveals a critical service gap with 3–6 years preschool education achieves almost full coverage, while institutional care for 0–3 year old remains underdeveloped (9). Recent policy initiatives, particularly the “Guiding Opinions on Promoting the Development of Childcare Services for Infants and Young Children Under 3 Years Old,” aim to address this disparity through rapid service expansion (4). In recent years, China has witnessed exponential growth in childcare facilities catering to children aged 0–3 years. The sector demonstrates a dual structure comprising public and private institutions, characterized by distinct service models. Public institutions primarily deliver subsidized childcare services through government support, while their private counterparts focus on premium offerings with enhanced educational resources and facilities, albeit at significantly higher costs. Current market analysis reveals private institutions dominate China’s childcare market share (4), creating an economic paradox where quality childcare accessibility becomes contingent upon families’ financial capacity. This commercialization trend raises two critical concerns: first, the prohibitive cost structure exacerbates household economic pressures, particularly for middle-income families; second, regulatory challenges emerge from private operators’ market fragmentation, manifesting in uneven service quality standards and operational transparency issues that complicate effective sectoral governance.

China’s childcare market development reveals systemic challenges that fall short of initial projections. Empirical studies identify a tripartite crisis in service provision, conceptualized as the 3A framework (accessibility, affordability, accountability) (10) The 3A issues significantly increase parents’ hesitation in choosing childcare services, leaving the enormous potential demand for childcare services in the market unsatisfied (5, 10, 11). The supply–demand imbalance in childcare services stems from supply-side optimizations failing to adequately address the complexity of demand. Childcare decisions among the childbearing-age population are dually influenced by objective conditions, such as economic status and family structure, and subjective perceptions, including parenting philosophies and service trust. Therefore, any effective supply-side reform must be based on a systematic analysis of these comprehensive demand drivers (12, 13).

However, the literature has scarcely explored how cognitive biases such as equating childcare with mere “babysitting” may suppress potential demand. Furthermore, while trust is a proven prerequisite for the utilization of various health services (14, 15), its role in shaping childcare demand remains largely unexamined. This study addresses this theoretical and empirical gap by constructing and testing a “Knowledge-Trust-Demand” model in Suzhou, a pioneering pilot city for China’s inclusive childcare policy (PUHUI childcare policy).

Existing research on childcare demand has predominantly focused on the “immediate” service choices of families with children, a perspective that is inherently retrospective. The innovation of this study lies in shifting the unit of analysis forward to the broader childbearing-age population (15–49 years) to capture their “potential” demand. For this demographic, the perceived accessibility of childcare is a critical consideration within their fertility decision-making process (1, 2). Therefore, focusing on the childbearing-age population not only allows for a more forward-looking prediction of future childcare needs but also provides key insights into the role of childcare services in broader population development strategies. Furthermore, most research has not differentiated childcare services by specific age groups of children, and no studies in China have specifically investigated influencing factors for childcare services targeting children aged 0–3 years. This study aims to analyze the current demand for childcare services for children aged 0–3 years among the childbearing-age population in Suzhou, China, to identify key influencing factors, and provide scientific evidence for enhancing childcare service quality and informing relevant policy formulation.

## Methods

### Participants and sample methods

A large-scale cross-sectional survey was conducted across multiple districts in Suzhou, China in August 2024. Suzhou was selected as the study site due to its status as a national pilot city for China’s inclusive (‘PUHUI’) childcare policy and its advanced economic development, providing a representative context for examining demand in a region with proactive policy implementation. Participants were selected using a multi-stage stratified random sampling method. The sample size was calculated based on the population distribution from the Seventh National Population Census in China, with a proportional sampling ratio of 0.05% of the total population in each district. To account for potential non-response and ineligible cases, the initial sample size was increased by 10%, resulting in a final expected target of 3,800 participants. Within each district, more than five townships or streets were randomly selected as primary sampling units. From these units, individuals of childbearing age (15–49 years) were systematically recruited by trained investigators. The survey team comprised healthcare professionals from local township/street health departments with expertise in maternal and child health services. Eligible participants were invited to complete a structured online questionnaire through a secure electronic data collection platform.

Participants were required to meet the following inclusion criteria: (1) aged between 15 and 49 years; (2) capable of independently understanding and completing the questionnaire in Chinese; and (3) proficient in using digital devices such as smartphones, tablets, or computers for online surveys. The exclusion criteria were as follows: (1) individuals aged under 15 or over 49 years; (2) those with communication or cognitive impairments that prevented them from independently understanding and completing the questionnaire; and (3) individuals unable to use digital devices to participate in the online survey.

### Measurement of variables

The survey systematically collected data across three domains: sociodemographic characteristics, household circumstances, and childcare service measurement.

Sociodemographic characteristics‌ captured eight key variables including age, gender, migration status, residence, education level, marital status, income and occupation. Migration status was defined as residing in Suzhou for more than six months without local household registration (hukou). Urban–rural residency was determined by current administrative address classification. Education status was categorized into five levels, ranging from junior high school or below to graduate degree or higher. Marital status included five options: unmarried, married with co-resident spouse, married with non-resident spouse, divorced/widowed, and others. Monthly income was stratified into five groups extending from less than 1,000 RMB to over 12,000 RMB. Occupational classification encompassed nine categories, including manager or head; professional technicians; civilian workers; business and service workers; agriculture, forestry, animal husbandry and fishery; production, transportation and manufacturing personnel; freelancing; unemployed; and others. Household circumstances‌ were assessed through four components. Participants reported their number of living children, cohabitation status with parents, total household members, and accessibility of childcare facilities within a 15-min walking radius.

The childcare service measurement‌ consisted of trust in childcare facilities, knowledge about childcare services, knowledge about childcare policy for children 0–3 years old and demand for childcare services. Institutional trustworthiness was measured using a 5-point Likert scale where 1 indicated “no trust” and 5 represented “complete trust.” Knowledge levels regarding existing childcare services and the childcare policy (specifically, the government-subsidized ‘PUHUI’ childcare policy, which encompasses financial subsidies, institutional quality standards, and service accessibility) were similarly assessed through 5-point scales, with higher scores denoting greater familiarity. The primary dependent variable - demand for childcare services - was dichotomously coded as 0 (no demand) or 1 (expressed need).

### Statistical analysis

All statistical analyses were conducted in STATA 17.0 through five methodological phases: descriptive statistics, chi-square tests, rank-sum tests, Pearson correlation analysis, and binary logistic regression. Descriptive analysis was used to describe the participants’ personal and family information, as well as their demand for childcare services. Continuous variables were expressed as medians with interquartile ranges, while categorical data were presented as frequencies with percentages. Chi-square tests (for categorical variables) and Wilcoxon rank-sum tests (for continuous/ordinal variables) were performed in univariate analysis to assess the associations between each independent variable and the dependent variable. Correlation analysis was used to assess the relationships among trust in childcare facilities, awareness of childcare services, awareness of inclusive childcare programs and childcare service demand. A binary logistic regression analysis was conducted with childcare services demand as the dependent variable and significant variables from univariate analysis as the independent variables, to identify the significant factors influencing the demand for childcare services.

## Results

### Descriptive analysis

Table 1 shows the characteristics of respondents. The survey achieved robust participation with 5,777 distributed questionnaires, of which 5,567 met validity criteria after quality control exclusions, resulting in a high response rate of 96.4%. The sample demonstrated pronounced gender disparity, with female predominance (80.7%, *n* = 4,489). Residential status distribution indicated 56.1% (*n* = 3,125) possessed local hukou registration, while nearly one-third (31.7%, *n* = 1,764) comprised interprovincial migrants. Notably, 81.6% (*n* = 4,543) of participants had established long-term residency exceeding six months in Suzhou, with 54.6% of respondents living in urban areas. Educational levels exceeded national averages, with 56.2% (*n* = 3,129) holding bachelor’s degrees or higher qualifications. 91.9% (*n* = 5,116) of respondents had entered legal unions, of whom 83.6% (*n* = 4,276) were living with their spouses. Economically, three-fifths of respondents (60.2%, *n* = 3,352) reported monthly earnings surpassing 4,000RMB, with labor force participation rates exceeding 60% across occupational categories. Household-level data demonstrated 86.0% (*n* = 4,788) of families included at least one child. Multigenerational cohabitation patterns emerged, with 58.1% (*n* = 3,233) residing with parent generations in households averaging four members. The spatial analysis revealed 44.7% (*n* = 2,491) could accurately identify childcare facility availability within a 15-min pedestrian catchment area. 52.7% of respondents expressed trust in childcare institutions. Almost one third of respondents knew about childcare services (70.5%) and childcare policy (63.9%) well. 45.9% of respondents expressed a demand for childcare services.

**Table 1:** The coding of variables and descriptive statistics (*N* = 5,567).

Variable	Definition and coding	Frequency	Percentage/Mean
Age	Participant age (years) in 2024	5,567	32.3
Gender	1 = Male	1,072	19.3%
	2 = Female	4,495	80.7%
Migrant	1 = Yes	1,412	25.4%
	2 = No	4,155	74.6%
Current residence	1 = Urban	2,527	45.4%
	2 = Rural	3,040	54.6%
Education	1 = Middle school or less	361	6.5%
	2 = High school or technical secondary school	620	11.1%
	3 = Junior college	1,458	26.2%
	4 = Bachelor	2,923	52.5%
	5 = Master or higher	205	3.7%
Marital status	1 = Unmarried	452	8.1%
	2 = Married & in the same city	4,654	83.6%
	3 = Married & not in the same city	359	6.5%
	4 = Divorced or widowed	67	1.2%
	5 = Others	35	0.6%
Income	1 = Less than 1000RMB	285	5.1%
	2 = 1001RMB - 4000RMB	1839	33.0%
	3 = 4001RMB - 8000RMB	2,710	48.7%
	4 = 8001RMB - 12000RMB	506	9.1%
	5 = More than 12000RMB	227	4.1%
Occupation	1 = Manager or head	113	2.0%
	2 = Professional technicians	524	9.4%
	3 = Civilian workers	932	16.7%
	4 = Business and service workers	467	8.4%
	5 = Agriculture, forestry, animal husbandry and fishery	32	0.6%
	6 = Production, transportation and manufacturing personnel	471	8.5%
	7 = Freelancing	1,010	18.2%
	8 = Unemployed	107	1.9%
	9 = Others	1911	34.3%
Number of children born	1 = no child	781	14.0%
	2 = one child	3,624	65.1%
	3 = two children	1,016	18.3%
	4 = three or more children	146	2.6%
Live with parents	1 = Yes	3,233	58.1%
	2 = No	2,334	41.9%
Family size	Number of people residing in the household	5,567	4
Childcare facility within a 15-min commute	1 = Yes	2,491	44.7%
	2 = No	1835	33.0%
	3 = Uncertain*	1,241	22.3%
Trust in childcare facilities	1 = Very distrusted	152	2.7%
	2 = Distrusted	346	6.2%
	3 = Medium	2,136	38.4%
	4 = Trusted	1828	32.8%
	5 = Very trusted	1,105	19.9%
Know about childcare services	1 = Not at all	427	7.7%
	2 = Not very well	1,216	21.8%
	3 = Moderately well	774	13.9%
	4 = Well	2,147	38.6%
	5 = Very well	1,003	18.0%
Know about PUHUI childcare policy	1 = Not at all	634	11.4%
	2 = Not very well	1,373	24.7%
	3 = Moderately well	1,082	19.4%
	4 = Well	1875	33.7%
	5 = Very well	603	10.8%
Demand for childcare services	0 = No need	3,013	54.1%
	1 = Need	2,554	45.9%

The “Uncertain” category indicates that respondents had unclear awareness of the presence of childcare facilities in their vicinity.

### Univariate analysis

Table 2 shows the results of univariate analysis. It indicates that there were significant differences in childcare services demand among different variable groups including gender, age, current residence, education level, marital status, monthly income, occupation, number of children, family size, and the presence of childcare facilities nearby (*p* < 0.05).

**Table 2:** The results of univariate analysis (*N* = 5,567).

Variables	Total	Demand for childcare services [n (%)]		*X*^2^/z	*P*
		No	Yes		
Gender				7.8954	0.005
Male	1,072	539 (50.3)	533 (49.7)		
Female	4,495	2,474 (55.0)	2021 (45.0)		
Age	5,567	32 (29–36)	32 (29–35)	−2.7270	0.006
Migrant				10.6499	0.001
Yes	1,412	817 (57.9)	595 (42.1)		
No	4,155	2,196 (52.8)	1959 (47.2)		
Current residence				20.9258	<0.001
Urban	2,527	1,283 (50.8)	1,244 (49.2)		
Rural	3,040	1730 (56.9)	1,310 (43.1)		
Education				60.3897	<0.001
Middle school or less	361	236 (65.4)	125 (35.6)		
High school or technical school	620	391 (63.1)	229 (36.9)		
Junior college	1,458	815 (55.9)	643 (44.1)		
Bachelor	2,923	1,475 (50.5)	1,448 (49.5)		
Master or higher	205	96 (46.8)	109 (53.2)		
Marital status				58.2627	<0.001
Unmarried	452	318 (70.4)	134 (29.6)		
Married and in the same city	4,654	2,429 (52.2)	2,225 (47.8)		
Married and not in the same city	359	203 (56.5)	156 (43.5)		
Divorced or widowed	67	41 (61.2)	26 (38.8)		
Others	35	21 (60.0)	14 (40.0)		
Income				18.8365	0.001
<1000RMB	285	168 (58.9)	117 (41.1)		
1,001-4000RMB	1839	1,041 (56.6)	798 (43.4)		
4,001-8,000RMB	2,710	1,447 (53.4)	1,263 (46.6)		
8,001-12,000RMB	506	256 (50.6)	250 (49.4)		
>12,000RMB	227	101 (44.5)	126 (55.5)		
Occupation				30.8289	<0.001
Manager or Head	113	58 (51.3)	55 (48.7)		
Professional technicians	524	230 (43.9)	294 (56.1)		
Civilian workers	932	489 (52.5)	443 (47.5)		
Business and service workers	467	268 (57.4)	199 (42.6)		
Agriculture, forestry, animal husbandry, fishery	32	17 (53.1)	15 (46.9)		
Production, transportation, manufacturing personnel	471	265 (56.3)	206 (43.7)		
Freelancing	1,010	547 (54.2)	463 (45.8)		
Unemployed	107	62 (57.9)	45 (42.1)		
Others	1911	1,077 (56.4)	834 (43.6)		
Number of children				466.9590	<0.001
No child	781	538 (68.9)	243 (31.1)		
One child	3,624	2,164 (59.7)	1,460 (40.3)		
Two children	1,016	263 (25.9)	753 (74.1)		
Three or more children	146	48 (32.9)	98 (67.1)		
Live with parents				1.4095	0.235
Yes	3,233	1728 (53.4)	1,505 (46.6)		
No	2,334	1,285 (55.1)	1,049 (44.9)		
Family size	4.12	4 (3–5)	4 (3–5)	11.6120	<0.001
Facility within a 15-min commute				138.6016	<0.001
Yes	2,491	1,164 (46.7)	1,327 (53.3)		
No	1835	1,018 (55.5)	817 (44.5)		
Uncertain	1,241	831 (67.0)	410 (33.0)		
Trust in childcare services	5,567	3 (3–4)	4 (3–5)	21.4920	<0.001
Know about childcare services	5,567	3 (2–4)	4 (3–4)	19.6740	<0.001
Know about childcare policy	5,567	3 (2–4)	4 (3–4)	16.1830	<0.001

Chi-square tests were performed for all categorical independent variables, and the results showed that gender differences exhibited substantial predictive power (*p* = 0.005), with male respondents showing a higher demand (59.7%) than female respondents (45%). Local residents (47.2%) showed a higher demand for childcare services than migrants (42.1%, *p* = 0.001), and the demand of urban residents (49.2%) was higher than rural residents (43.1%, *p* < 0.001). The demand increased with higher respondents’ education level and income (*p* < 0.01). There were significant differences across marital statuses (*p* < 0.001), with married couples who lived in the same city showed the highest childcare services demand (47.8%) and unmarried respondents showed the lowest demand (29.6%). Respondents in different occupations showed different demands significantly (*p* < 0.001), with the highest demand for professional technicians (56.1%) and the lowest demand for unemployed people (42.1%). The demand increases with the size of the family (*p* < 0.001), with the families having two children showing the highest demand (74.1%). Demand was significantly higher among respondents who knew there was a childcare provider within a 15-min radius (53.3%) than that of those who did not know (44.5%) or uncertain (33%).

Wilcoxon rank-sum tests were performed for the continuous variables of age, trust in childcare facilities, know about childcare services, and know about childcare policy. The results showed that the older respondents had lower demand for childcare services (*z* = 2.7270, *p* = 0.006). The demand of respondents who had higher levels of trust in childcare facilities (*z* = 21.4920, *p* < 0.001), know about childcare services (*z* = 19.6740, *p* < 0.001) and know about childcare policy was higher (*z* = 16.1830, *p* < 0.001) (Table 3).

**Table 3:** The results of binary logistic regression (*N* = 5,567).

Variables	Coefficient	SD	*P*	OR (95%CI)
Gender (Ref. = Male)				
Female	−0.399	0.077	<0.001	0.671 (0.577–0.781)
Age	−0.051	0.007	<0.001	0.950 (0.938–0.963)
Migrant (Ref. = Yes)				
No	0.063	0.073	0.384	1.065 (0.924–1.229)
Current residence (Ref. = Urban)				
Rural	−0.254	0.063	<0.001	0.776 (0.685–0.878)
Education (Ref. = Middle school and below)				
High school or technical school	0.195	0.151	0.197	1.215 (0.904–1.634)
Junior college	0.295	0.137	0.031	1.344 (1.027–1.758)
Bachelor	0.389	0.137	0.005	1.476 (1.127–1.932)
Master or higher	0.721	0.212	0.001	2.057 (1.358–3.115)
Marital status (Ref. = Unmarried)				
Married and in the same city	0.217	0.167	0.195	1.242 (0.895–1.724)
Married and not in the same city	0.230	0.196	0.240	1.259 (0.857–1.849)
Divorced or widowed	−0.374	0.326	0.251	0.688 (0.364–1.302)
Others	0.383	0.408	0.347	1.467 (0.659–3.265)
Monthly income	−0.068	0.040	0.086	0.934 (0.864–1.010)
Occupation (Ref. = Manager or Head)				
Professional technicians	0.387	0.232	0.095	1.472 (0.935–2.318)
Civilian workers	0.027	0.223	0.903	1.028 (0.664–1.590)
Business and service workers	−0.069	0.237	0.771	0.933 (0.587–1.434)
Agriculture, forestry, animal husbandry and fishery	0.376	0.446	0.400	1.456 (0.607–3.493)
Production, transportation and manufacturing personnel	0.245	0.238	0.302	1.278 (0.802–2.036)
Freelancing	0.121	0.225	0.589	1.129 (0.727–1.754)
Unemployed	0.352	0.308	0.253	1.422 (0.777–2.601)
Others	−0.030	0.218	0.890	0.970 (0.633–1.486)
Number of children born (Ref. = No child)				
One child	0.519	0.138	<0.001	1.680 (1.282–2.202)
Two children	1.609	0.155	<0.001	4.999 (3.688–6.775)
Three or more children	1.380	0.236	<0.001	3.979 (2.504–6.321)
Family size	0.007	0.025	0.767	1.007 (0.959–1.058)
Childcare facility within a 15-min commute (Ref. = Uncertain)				
Yes	−0.170	0.088	0.054	0.844 (0.710–1.003)
No	0.138	0.085	0.104	1.148 (0.972–1.356)
Trust in childcare services	0.468	0.039	<0.001	1.596 (1.479–1.722)
Know about childcare services	0.204	0.035	<0.001	1.226 (1.146–1.312)
Know about childcare policy	0.082	0.033	0.013	1.085 (1.017–1.157)

### Multivariate analysis

A binary logistic regression model was constructed to determine the influencing factors of childcare service demand, with significant variables in chi-square tests and rank-sum test as independent variables. Variables including gender, age, current residence, education level, number of children, trust in childcare services, know about childcare services and childcare policy had significant influence on respondents’ childcare service demand (*p* < 0.05).

The demand for childcare services increased significantly with trust in childcare institutions, know about childcare services and childcare policy, but decreased with age (*p* < 0.05). Notably, female respondents demonstrated lower childcare service demand than male (OR = 0.671, *p* < 0.001), and the demand of rural residents was lower than urban residents (OR = 0.776, *p* < 0.001). The demand of respondents who had a junior college education level or above was higher than those who had middle school and below education level (*p* < 0.05). The respondents who had two children had the highest demand (OR = 4.999, *p* < 0.001).

## Discussion

In response to China’s persistent demographic challenges, the Chinese government has implemented comprehensive policy interventions since 2020 to revitalize birth rates through enhanced childcare service support (1, 10, 16). However, these initiatives continue to confront the entrenched the “3A” dilemma - encompassing availability, affordability, and accessibility - within China’s childcare infrastructure (10). To better understand the demand-side factors influencing the achievement of these policy goals, we conducted a cross-sectional study investigating childcare service demand for infants aged 0–3 years old and its influencing factors among reproductive-aged individuals in Suzhou, China. The results indicated that less than half of respondents reported active demand for childcare services for infants aged 0–3 years old.

Our analysis identified significant gender-based disparities in childcare service demand for 0–3 years old infants, where males showed higher demand than females. Previous studies demonstrated inconsistent results, which suggested that women’s heightened demand for professional childcare stems from‌ pronounced work–family conflicts (4, 11, 17). However, when addressing childcare for children under the age of three, our findings suggest that the entrenched gendered division of labor continues to significantly influence parental caregiving preferences. This is reflected in the tendency for mothers to predominantly opt for personal caregiving or relying on intergenerational support, a choice shaped not only by cultural emphasis on maternal nurturing but also by structural barriers in the workforce. In contrast, fathers generally demonstrate greater propensity to delegate childcare responsibilities to professional institutions. This gendered division of childcare labor enables male caregivers to allocate additional time to work commitments while adhering to conventional family role perceptions. In addition, previous researches have shown that women have high requirements for safety conditions, distance from home, and teachers’ competencies, and they were not comfortable leaving children under the age of two in childcare facilities out of concerns for children (4). Regarding gender differences, the key lies in enhancing the quality and flexibility of childcare services to better meet women’s high expectations and concerns, such as improving safety conditions, providing convenient locations, and ensuring that teachers are competent. In addition, more publicity and education should be carried out to change the traditional view of family parenting and liberate more labor.

Previous research has indicated that older individuals are more likely to opt for childcare services due to factors such as limited energy (4, 18, 19). Contrary to previous findings that older caregivers demonstrate elevated service utilization rates, our analysis revealed older individuals showed lower demand. One practical explanation is that the older respondents may have completed their family planning, reducing their need for childcare services, particularly for three-year-old children. Beyond this, the result can be interpreted through the lens of intergenerational differences in caregiving ideologies. Older cohorts, socialized when traditional, family-based caregiving was the norm, may perceive formal institutional services with greater skepticism regarding their necessity and safety. This perspective is consistent with existing research highlighting generational shifts in parenting beliefs and reliance on family support structures (20–22). Although the cross-sectional data of this study cannot directly test this causal pathway, it highlights a crucial direction for future research: a dedicated comparative analysis of childcare preferences among different age cohorts of the childbearing-age population.

Rural residents demonstrated lower childcare service utilization probabilities than urban counterparts. This may be because rural residents are more likely to live with their parents and have relatively lower social and economic status (4, 23). The cost of institutional care remains higher than that of parental or grandparent care in rural areas. Furthermore, compared to urban centers, rural areas likely face significant gaps in childcare service coverage, accessibility such as the availability of institutions within a 15-min commute. This undersupply can directly suppress the formation and expression of potential demand. Another reason is that farmers have flexible schedules, allowing them to take care of infants and young children aged 0–3. The Chinese government should consider the current urban–rural divide and increase the level of subsidies for childcare services in rural areas.

‌Respondents with lower educational attainment exhibited diminished demand for childcare services targeting children aged 0–3 years. This pattern may stem from the dual mechanisms of socioeconomic stratification and informational disparities: individuals with limited formal education typically occupy lower socioeconomic positions, while simultaneously demonstrating reduced awareness of both childcare service availability and associated policies (19, 24, 25). Targeted efforts should focus on enhancing awareness and comprehension of childcare services among demographically vulnerable groups, particularly older adults and individuals with limited educational attainment. Implementing tailored public outreach initiatives and community-based educational interventions that highlight the dual developmental advantages for both children and parental well-being proves essential in addressing this need.

Our study further revealed that family characteristics had a significant impact on childcare demand. Child-rearing individuals demonstrated substantially greater need for childcare support compared to their childless counterparts, with service requirements exhibiting a positive correlation with offspring quantity. The care of multiple infants heightens caregiving complexity, necessitating greater parental time and energy investment, thereby amplifying dependence on professional childcare services (4, 24). Building on these findings, we propose a dual-pronged policy framework to address identified determinants. For families with multiple children, governments should implement progressive subsidy systems weighted by family size, coupled with tax relief measures to mitigate childcare-related financial pressures. Concurrently, strategic investment should focus on capacity expansion and quality enhancement in high-demand regions through infrastructure development and workforce training programs.

The childcare service-related indicators also demonstrated significant associations with childcare service demand. Specifically, trust in childcare facilities was found to markedly enhance the demand for childcare services, which was consistent with previous studies (26, 27). Furthermore, parental knowledge about childcare services significantly predicted demand, corroborating prior findings (27). When interpreting the positive association between knowledge and demand, potential reverse causality must be cautiously considered. Specifically, a strong pre-existing need for childcare may itself motivate individuals to more actively seek information about services and policies, rather than knowledge unilaterally generating demand. Despite this potential bidirectionality, this study’s innovative finding—that awareness of childcare policies for infants aged 0–3 significantly enhances demand likelihood—remains valuable. This suggests that effective policy communication can lower perceived barriers and thereby promote service engagement. Notably, even accounting for demand-driven information seeking, nearly one-third of respondents in our study lacked awareness of childcare services and related policies, clearly indicating a significant gap and room for improvement in current information dissemination efforts. Multiple measures should be implemented to enhance the trust of childbearing-age populations in childcare institutions, raise their awareness of childcare services, and improve their understanding of childcare policies. First‌, operational transparency and quality assurance of childcare institutions are crucial. Standards for the establishment and operation of childcare institutions should be elevated by establishing a quality management system compliant with ISO 9001 standards. Operational information, including staff qualifications, safety records, and curriculum standards, should be disclosed in real time through public dashboards. Additionally, a collaborative regulatory system involving government agencies, parents, media, and civil society should be established and the accessible oversight channels should be ensured and smoothed. Second‌, efforts to enhance public awareness of childcare services and related policies must be intensified. Community-based policy interpretation sessions should be organized to improve residents’ understanding of the benefits of childcare services. Leveraging new media platforms such as WeChat public platform and Douyin (TikTok) for targeted campaigns will amplify the visibility of childcare services and associated subsidy policies. Third‌, the government should implement a certification and evaluation program for childcare institutions, incorporating financial incentives for accredited providers. This initiative will not only elevate service quality and standardization but also foster parental trust in childcare institutions, thereby creating a virtuous cycle of quality improvement and demand stimulation.

### Limitations

This study has several limitations. First, the dichotomous measurement of childcare service demand (need/no need) may not capture the full spectrum of demand intensity or type; future research could employ more nuanced, multi-dimensional indicators. Second, the cross-sectional design precludes definitive causal inferences, particularly concerning knowledge-related variables (‘Know about childcare services,’ ‘Know about childcare policy’) which may exhibit endogeneity due to reverse causality; longitudinal studies are warranted to clarify these relationships. Third, reliance on an online survey might introduce sampling bias by potentially underrepresenting individuals with lower digital literacy or access, and self-reported data are susceptible to social desirability and recall biases. Finally, the Suzhou-specific sample limits the generalizability of findings to other regions in China. As an economically advanced region and a pioneer in implementing national childcare policies, Suzhou’s context may not be representative of less developed areas or regions without similar policy support. Broader geographical sampling is recommended for future studies to enhance the generalizability of the findings.

## Conclusion

This study systematically identified the key determinants of demand for childcare services for infants aged 0–3 among China’s childbearing-age population. Empirical findings reveal that sociodemographic characteristics, including gender, age, urban–rural residence, educational attainment, and parity—as well as subjective cognitive factors, such as trust in childcare institutions and awareness of services and policies, are significant predictors of demand.

Based on these findings, to effectively enhance the utilization of childcare services, this study proposes two core policy recommendations: Focus on vulnerable groups to bridge the urban–rural and educational divides. The lower demand observed among women, older individuals, rural residents, and those with lower educational attainment points to structural barriers in service accessibility and acceptability. Therefore, policies should prioritize rural and underserved areas, aiming to precisely lower childcare barriers for these groups through optimized facility distribution and targeted financial subsidies. Strengthen trust-building and information empowerment to unlock the potential of multi-child families. Trust and awareness are powerful drivers of demand, while multi-child families face higher pressure despite their greater need. Therefore, the government should adopt a two-pronged approach: on one hand, build public trust through rigorous industry standards and transparent regulatory mechanisms; on the other, launch extensive public education campaigns to promote the value of formal childcare and ensure that preferential or subsidy policies for multi-child families are accurately communicated and easily accessible.

## Data Availability

The raw data supporting the conclusions of this article will be made available by the authors, without undue reservation.
